# MCL-1 is a clinically targetable vulnerability in breast cancer

**DOI:** 10.1080/15384101.2022.2054096

**Published:** 2022-03-29

**Authors:** Matthew L Winder, Kirsteen J Campbell

**Affiliations:** a CRUK Beatson Institute, Garscube Estate,Switchback Road, Glasgow, G61 1BD, UK; b Institute of Cancer Sciences, University of Glasgow, Garscube Estate, Switchback Road, Glasgow, G61 1QH, UK

**Keywords:** MCL-1, apoptosis, breast cancer, BH3-mimetics, chemotherapy

## Abstract

Pro-survival members of the BCL-2 family, including MCL-1, are emerging as important proteins during the development and therapeutic response of solid tumors. Notably, high levels of MCL-1 occur in breast cancer, where functional dependency has been demonstrated using cell lines and mouse models. The utility of restoring apoptosis in cancer cells through inhibition of pro-survival BCL-2 proteins has been realized in the clinic, where the first specific inhibitor of BCL-2 is approved for use in leukemia. A variety of MCL-1 inhibitors are now undergoing clinical trials for blood cancer treatment and application of this new class of drugs is also being tested in solid cancers. On-target compounds specific to MCL-1 have demonstrated promising efficacy in preclinical models of breast cancer and show potential to enhance the anti-tumor effect of conventional therapies. Taken together, this makes MCL-1 an extremely attractive target for clinical evaluation in the context of breast cancer.

**Abbreviations:** ADC (antibody-drug conjugate); AML (Acute myeloid leukemia); APAF1 (apoptotic protease activating factor 1); bCAFs (breast cancer associated fibroblasts); BCL-2 (B-cell lymphoma 2); BH (BCL-2 homology); CLL (chronic lymphocytic leukemia); EGF (epidermal growth factor); EMT (epithelial to mesenchymal transition); ER (estrogen receptor); FDA (food and drug administration); GEMM (genetically engineered mouse model); HER2 (human epidermal growth factor 2); IL6 (interleukin 6); IMM (inner mitochondrial membrane); IMS (intermembrane space); MCL-1 (myeloid cell leukemia-1); MOMP (mitochondrial outer membrane permeabilisation); MM (multiple myeloma); PDX (patient-derived xenograft); OMM (outer mitochondrial membrane); PROTAC (proteolysis-targeting chimeras) TNBC (triple negative breast cancer); UPS (ubiquitin mediated proteolysis system)

## Introduction

Survival of patients diagnosed with breast cancer has improved dramatically in recent decades, largely owing to early detection through extensive screening programs, refinement of conventional treatments, and the advent of effective targeted and personalized therapies. Although there has been a ~ 40% decline in death rates of female breast cancer patients since the 1980s, the decreasing rate of mortality has slowed and it is estimated that global incidence will continue to rise steadily over the next 10–20 years [1,2]. Worldwide, breast cancer is the most prevalent tumor type and remains the leading cause of cancer-related death amongst women, highlighting the essential requirement for improved treatment strategies [2]. During progression to lethality, solid tumors often overcome a series of key evolutionary bottlenecks including: primary tumor establishment; metastatic dissemination and seeding; and resistance to therapeutic intervention, and evasion of cell death is a prerequisite for survival in the presence of each of these selection pressures [3].

Cell death can occur through several mechanisms but apoptosis, a form of regulated cell death, is the mechanism that is most commonly perturbed in cancer. Deregulation of processes that control intrinsic (mitochondrial) apoptosis is prominent in both tumorigenesis and during failed responses to cancer therapy [4]. Activation of mitochondrial outer membrane permeabilisation (MOMP), a key event in intrinsic apoptosis, is governed by interactions within the multi-member BCL-2 (B-Cell Lymphoma 2) family of proteins. First characterized in hematological cancers, it is now recognized that alterations in the BCL-2 family are also frequent in solid tumors and that failed intrinsic (mitochondrial) apoptosis facilitates tumor survival throughout the oncogenic process [4,5].

Here, we focus on MCL-1 (Myeloid Cell Leukemia-1), a pro-survival BCL-2 family protein best characterized for its role in hematopoiesis and related malignancies of the blood. MCL-1 is required in many cell types as demonstrated by the embryonic lethality of germline *Mcl1* deletion in mice, whilst conditional genetic ablation revealed a functional requirement of MCL-1 for survival of specific cell types such as hematopoietic stem cells and cardiomyocytes [6–8]. It is now understood that *MCL1* is frequently upregulated across a range of solid tumor types, including breast cancer [9]. Importantly, dependency on MCL-1 in cancer cells may be exploited for therapeutic gain, and novel agents that specifically target MCL-1 are already undergoing clinical investigation as potential chemotherapeutics in acute myeloid leukemia (AML) and multiple myeloma (MM). Whilst elevation of MCL-1 may bestow a competitive advantage on breast cancer cells during tumor development and prevent tumor cell elimination in response to therapy, the reliance on MCL-1 for survival also represents a vulnerability that could be utilized to improve treatment outcome in breast cancer.

## Induction of intrinsic apoptosis is controlled by the BCL-2 family

BCL-2 was first identified through mapping of the t[12:18] chromosomal translocation, which results in a constitutive BCL-2 expression from the immunoglobulin locus and often underpins follicular lymphoma [10–12]. Initially, BCL-2 was considered functionally unique amongst known tumor-promoting genes as it supports oncogenesis by preventing tumor cell death rather than driving tumor cell proliferation [13,14]. Numerous proteins with regions of sequence homology to BCL-2 have since been identified and characterized as part of the BCL-2 family, and these fall into two categories with opposing pro-apoptotic and pro-survival functions [15]. Protein–protein interactions between BCL-2 family members determine whether a cell undergoes apoptosis, and the relative quantity and activity of these proteins fluctuates throughout mammalian development to enable differential states of susceptibility to pro-apoptotic stimuli. The propensity of a cell to undergo apoptosis following the receipt of a stress stimulus is described as the extent to which it is “primed” [16].

**Figure 1.**
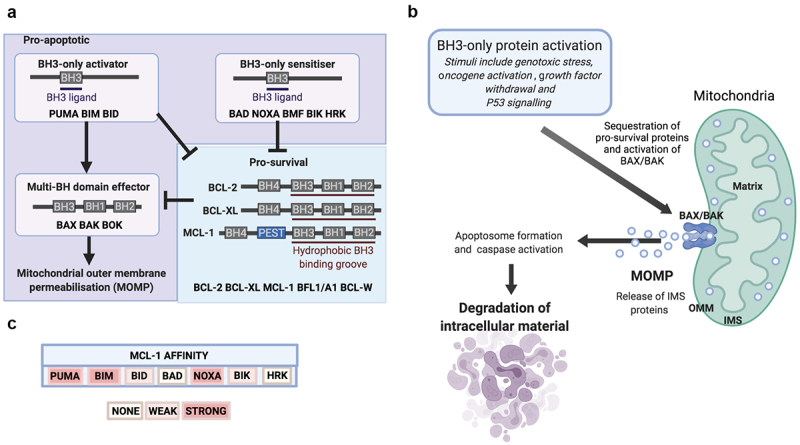
BCL-2 family and intrinsic (mitochondrial) apoptosis. (**A**) Mitochondrial outer membrane permeabilisation (MOMP) is controlled by protein–protein interactions between members of the BCL-2 family. (**B**) Intrinsic apoptosis is initiated by internal or external stress stimuli such as DNA damage, oncogene activation or cytokine deprivation. These events activate intracellular signalling pathways which manifest by altering the balance of pro-apoptotic and pro-survival BCL-2 family proteins. In response to sufficiently toxic stress stimuli, pro-apoptotic BCL-2 family proteins are activated and overwhelm pro-survival proteins to liberate BAX/BAK, which initiates MOMP and the release of intermembrane space (IMS) proteins into the cytosol to activate apoptosome formation, caspase activation and degradation of intracellular material. (**C**) Members of the BCL-2 family of proteins bind to each other with highly selective affinity, and the electropositive surface within the BH3 binding groove of MCL-1 mediates strong affinity for the BH3-only proteins PUMA, BIM, and NOXA, but not BAD.

BCL-2 family proteins share at least one of four highly conserved BCL-2 homology (BH) regions. The pro-apoptotic BCL-2 family members can be subdivided into BH3-only proteins, so-called because they possess only the conserved alpha-helical BH3 domain, and multi-BH domain “effector” proteins, such as BAX/BAK (Figure 1A). Specifically, BH3-only “sensitizer” proteins (including BAD and NOXA) can indirectly enhance apoptosis through binding and occupying pro-survival proteins to ameliorate inhibitory sequestration of BAX/BAK [17,18]. As well as undertaking this function, BH3-only “activator” proteins (including BIM, PUMA, and BID) can bind directly to BAX/BAK to facilitate apoptosis [17,18] (Figure 1A).

This process is antagonized by multi-BH domain pro-survival BCL-2 proteins (such as BCL-2, BCL-XL, and MCL-1) which maintain mitochondrial integrity through binding and sequestering pro-apoptotic members of the BCL-2 family (Figure 1A). Structural studies reveal that these associations are mediated by a hydrophobic binding groove (composed of BH1-3) which guides the interaction with hydrophobic residues of exposed alpha-helical BH3 domains [19,20]. The culmination of successful pro-apoptotic signaling is the activation of BAX/BAK, which oligomerize to form macropores and permeabilise the outer mitochondrial membrane (OMM) (Figure 1). Mitochondrial outer membrane permeabilization (MOMP) enables the release of mitochondrial intermembrane space (IMS) proteins, such as cytochrome *c*, OMI and SMAC into the cytoplasm. Subsequently, cytochrome *c* can then complex with cytosolic apoptotic protease activating factor 1 (APAF-1) to form the apoptosome, a multimolecular protein complex, and this drives activation of the caspase-9/7/3 (cysteine-aspartic protease) cascade to trigger the destruction of cellular contents [21–25] (Figure 1B).

## Interaction with BH3-only proteins regulates MCL-1 activity

The sequence of *MCL1* is most divergent from *BCL2* amongst the pro-survival BCL-2 family members, as homology occurs only in the defined BH domains [26,27]. Much like other pro-survival BCL-2 family proteins, the BH1-3 domains of MCL-1 form a conserved hydrophobic groove that mediates interactions with the hydrophobic BH3 face of certain pro-apoptotic BCL-2 family members (Figure 1C) [28–30]. Distinct pro-survival BCL-2 family proteins have differential affinity for specific BH3 domain-containing pro-apoptotic proteins, and this is promoted by the unique amino acid residues that constitute the central alpha-helices. The electropositive surface within the BH3 binding groove of MCL-1 mediates a selective affinity for critical residues within the BH3 domain of BAK, BIM, BID, NOXA, and PUMA, but an inability to interact with BAD (Figure 1C) [17,18,31]. The binding interaction between MCL-1 and NOXA triggers proteasomal degradation of MCL-1, whereas binding to PUMA or BIM serve to stabilize MCL-1 in an inactive state without stimulating protein degradation [30,32,33]. The BH3-only sensitizer protein NOXA is highly selective for MCL-1, and NOXA expression efficiently induces apoptosis in cells manipulated into MCL-1 dependency [34]. Interestingly, high expression of *NOXA* mRNA has been shown to associate with improved survival in breast cancer and predict response to microtubule targeting chemotherapeutic agents [35]. Furthermore, resistance to targeted therapies in *HER-2* amplified breast cancer can be conferred by microRNA-4728-mediated suppression of *NOXA* which serves to prevent apoptosis of breast cancer cells in an MCL-1 dependent manner [36]. Taken together, this highlights the intimate relationship between MCL-1 protein levels and NOXA expression which may be important for progression and drug resistance in breast cancer patients.

## MCL1 amplification and expression in breast cancer

**Figure 2.**
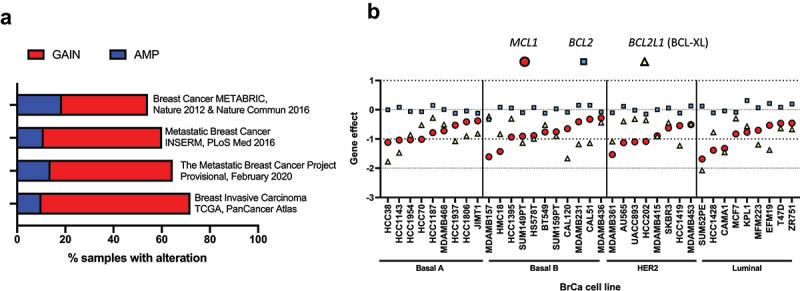
MCL-1 importance in breast cancer patients and breast cancer cell lines. (**A**) *MCL1* is frequently increased in breast tumours. Genomic gain (red) or amplification (blue) of *MCL1* occurs in up to 72% of breast cancer samples assayed for copy number alteration in non-redundant datasets composed of greater than 100 samples curated in cBioportal [39–43]. **(B)** Essentiality of pro-survival BCL-2 family members in breast cancer. This graph illustrates the functional importance of pro-survival members of the BCL-2 family in a compendium of “CRISPR knockout screens” across breast cancer cell lines from the Cancer Cell Line Encyclopaedia (CCLE). TNBC cell lines are often split into Basal A and B subgroups, where Basal A represents a tumour subtype which is more representative of basal-like tumours and Basal B is increasingly claudin-low and stem/mesenchymal -like. A lower gene effect score indicates an increased likelihood that the gene is essential in a given cell line. A gene effect score of 0 infers non-essentiality, whereas a gene effect score of −1 represents the median of all pan-essential genes (https://depmap.org/portal/) DepMap 21Q2 dataset [123].

Genomic gain of chromosome 1q21.2, where *MCL1* is located, is frequent across a range of tumor types and is particularly common in breast cancers [9]. Breast cancer encompasses a spectrum of tumor subtypes with distinct morphological features, biological properties, and clinical implications. These can be broadly stratified by molecular and histopathological criteria including estrogen and progesterone receptor (ER/PR) status, amplification of *ERBB2* (commonly referred to as human epidermal growth factor 2 [HER2]), and the transcriptomic landscape. The resultant classification system remains centered on the “classical” ER-positive /PR-positive, *HER2*-amplified and triple-negative breast cancer (TNBC) subtypes in the clinic, and this commonly informs the current therapeutic standard of care. However, breast cancer can be molecularly divided into a further series of subtypes including luminal A and B, *HER2*-enriched, basal-like, and claudin-low [37,38]. Amplification/gain of *MCL1* persists across breast cancers independent of the tumor stratification approach and is observed in up to 72% of breast cancer cases in online genomic data repositories (Figure 2) [39–43]. Notably, the frequency of *MCL1* amplification and mRNA expression exceeds both BCL-2 (~9%) and BCL-XL (~27%) across all major subtypes of breast cancer, suggestive of a specific importance of MCL-1 in breast tumourigenesis [39–43].

MCL-1 protein abundance is the product of a series of upstream events, which may also be perturbed in breast cancer, such that quantification of *MCL1* gene and mRNA copy number levels may underestimate the extent to which MCL-1 is upregulated [44]. In a study of 428 treatment naïve breast cancers we observed a range of MCL-1 protein expression within all classical subtypes, and each subtype contained cases possessing high levels of MCL-1 [45]. High MCL-1 protein expression was significantly associated with poor prognosis, increased invasive grade and lymph node positivity [45]. Interestingly, elevated MCL-1 protein was predictive of poor outcome in both TNBC and non-TNBC patients but did not correlate with outcome in the *HER2*-amplified subtype, although HER2 positivity was correlated with increased MCL-1 expression [45]. These studies support the widely available genomic and transcriptomic data detailing elevated levels of MCL-1 in breast cancer, and suggest that increased MCL-1 expression confers a selective advantage during breast tumorigenesis. It has been demonstrated using matched pairs of ER-positive breast cancers that the level of *MCL1* alteration in primary and metastatic disease is comparable, so it remains to be determined whether MCL-1 expression is important for metastatic dissemination or if increases in MCL-1 protein occur at secondary tumor sites [46].

The elevation of MCL-1 during tumor development may also impart an intrinsic resistance to cancer therapy. Indeed, high MCL-1 protein levels were indicative of poor response to a paclitaxel-containing treatment regime in a cohort of invasive breast cancer patients [47]. Therapeutic resistance may also be acquired through alteration in *MCL1* expression during treatment, and molecular profiling of post-adjuvant chemotherapy TNBC patient tumors revealed amplification of *MCL1* in 54% of cases and showed that *MCL1* amplification can arise during treatment [48]. Furthermore, analysis of paired TNBC biopsy samples pre-/post-chemotherapy showed that MCL-1 protein levels were increased after treatment, suggesting that MCL-1 may become further up-regulated in response to chemotherapy [49].

## Transcriptional control of MCL-1 expression can be targeted for therapeutic effect in breast cancer

**Figure 3.**
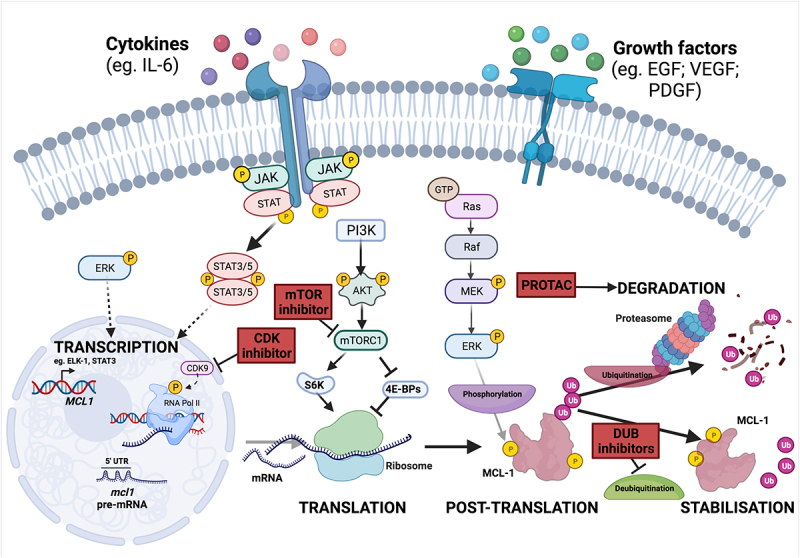
Regulation of MCL-1 and mechanisms to reduce MCL-1 levels. *MCL1* expression is stimulated following the receipt of diverse extracellular cytokines and growth factors. The signalling pathways which are predominantly responsible for inducing MCL-1 transcription, translation and protein stability include the PI3K/AKT pathway, JAK/STAT signalling and the MEK/ERK cascade. *MCL1* mRNA is highly unstable and is particularly sensitive to widespread change in the rate of gene expression, protein synthesis and protein degradation. Potential pharmacological methods to decrease MCL-1 levels in cancer are indicated in red boxes. Inhibition of transcription-associated cyclin-dependent kinases (CDKs), such as CDK9, leads to decreased levels of short-lived mRNA and proteins, including MCL-1, and CDK inhibitors are undergoing clinical trials. MCL-1 protein normally has a short half life, and levels are strongly impacted by changes in the rate of translation. Inhibitors of mTOR may represent a viable approach to suppress cap-dependent translation and thereby reduce MCL-1 protein. MCL-1 stability can also be targeted by proteolysis-targeting chimeras (PROTACs) which selectively bind a target protein and recruit an E3 ligase to initiate proteosomal degradation. A series of deubiquitinases (DUBs) have been reported to remove ubiquitin moieties and thus stabilise MCL-1, and DUB inhibitors could also have the potential to prevent MCL-1 deubiquitination and thereby reduce MCL-1 protein levels.

MCL-1 abundance is controlled at multiple levels including transcriptional, translational, and post-translational mechanisms, and each of these could be therapeutically targeted to reduce MCL-1 levels in breast cancer (Figure 3). *MCL1* was first identified as an immediate-early gene transcriptionally up-regulated in the ML-1 human myeloid leukemia cell line upon phorbol-ester induced differentiation [26]. Signaling through MEK/ERK has been shown to induce transcription of *MCL1* via ELK-1 and SRF transcription factors, and this pathway is commonly dysregulated during breast tumor development [50,51]. Various other signaling pathways and transcription factors have been implicated in stimulating *MCL1* expression in response to diverse cytokines, growth factors, and stress stimuli, such as hypoxia, oncogene activation, and endoplasmic reticulum (ER) stress [52]. For example, it has been illustrated that pro-inflammatory cytokine interleukin (IL)-6 potently stimulates JAK/STAT signaling in breast cancer cell lines, and it has also been shown that both IL-6 and JAK/STAT signaling can potentiate *MCL1* transcription in multiple cancer subtypes [53–56]. These mechanisms could be important in breast cancer where STAT3 is often constitutively active, and it has also been shown that higher levels of IL-6 in the blood of metastatic breast cancer patients correlates with poor prognosis [57,58].

Cyclin-dependent kinase (CDK) inhibitors have been shown to down-regulate *MCL1* transcription, and decreased MCL-1 expression is necessary for these agents to induce apoptosis. Whilst most CDKs primarily function through mediating cell-cycle transition, CDK7/8/9 are critical for transcription by RNA polymerase II. Indeed, inhibition of CDKs with dinaciclib has demonstrated promising clinical efficacy in pre-clinical models of lymphoma and *HER2*-amplified breast cancers [59,60]. This process is reliant upon inhibition of *MCL1* transcription, liberation of pro-apoptotic BAK and subsequent BAK-induced mitochondrial apoptosis [59,60]. Interestingly, targeted CDK9 inhibitors, such as voruciclib, potently induce apoptosis through MCL-1 depletion in many hematologic cell lines and in a subset of breast cancer cell lines, and have recently entered clinical trials for the treatment of hematological malignancies (Figure 3) [61,62]. Indirect targeting of MCL-1 through CDK9 inhibition potently sensitizes non-Hodgkin Lymphoma and AML cells to the BCL-2 inhibitor venetoclax without significant toxicity to mice suggesting that this strategy to co-target multiple pro-survival BCL-2 proteins could be tolerable if applied to breast cancer in the clinic [61,63].

## Translational control can be leveraged to reduce MCL-1

MCL-1 is a particularly short-lived protein (estimated half life ~1–3 hours) and *MCL1* mRNA is considered “weak” due to its GC-rich and highly structured 5’ untranslated region (UTR) [64,65]. Taken together, this conspires to preferentially sensitize MCL-1 to alterations in the rate of global protein synthesis and offers the opportunity to indirectly target MCL-1 levels [66]. As well as driving MEK/ERK-mediated phosphorylation of ELK-1 to activate *MCL1* transcription, epidermal growth factor (EGF) induces PI3K pathway activation to stimulate mammalian target of rapamycin (mTOR) regulated cap-dependent translation, and inhibition of mTOR complex 1 (mTORC1) sensitizes multiple cancer cell lines and mouse models to apoptosis through decreased MCL-1 protein levels [50,65,67–69]. Further to this, EGF signaling can also control MCL-1 protein abundance through mTOR-mediated phosphorylation and activation of ribosomal protein S6 kinase (S6K), a critical enzyme for ribosome recruitment during mRNA translation. Interestingly, *Mcl1* deficiency impairs lactogenesis and delays reconstitution of mammary glands by mammary stem and progenitor cells *in vivo*, and independent inhibition of both EGF and mTOR serves to recapitulate this phenotype alongside an associated decrease in MCL-1 levels [70].

These mechanisms may also be important in breast cancer as a series of breast cancer cell lines undergo cap-dependent translational increase in MCL-1 after BCL-2 and/or BCL-XL inhibition, and it has also been illustrated that a translational increase in MCL-1 protein can occur through the mTOR/4E-BP axis in *PIK3CA*-mutant breast cancers [71]. Importantly, increased MCL-1 protein levels through PI3K/AKT signaling can enhance MCL-1 pro-survival functionality in the absence of genomic and transcriptomic *MCL1* upregulation, indicating that targeting mTOR-mediated MCL-1 translation may provide an additional mechanism by which MCL-1 protein levels can be suppressed in some breast cancer subtypes (Figure 3) [71]. Enhanced translation of *MCL1* may be a common feature in ER-positive breast cancer where ERα has been shown to bind RNA and increase translation of specific mRNAs, including *MCL1*, to enhance cell survival and resistance to tamoxifen [44].

## Control of MCL-1 protein stability

MCL-1 is the largest pro-survival BCL-2 family protein due to the presence of an extended N-terminus rich in PEST (proline (P), glutamic acid (E), serine (S), and threonine (T)) motifs (Figure 1A) [26,72]. PEST sequences commonly act as a scaffold for post-translational modifications, including phosphorylation and ubiquitination, and this facilitates the rapid turnover of MCL-1 protein when compared to BCL-2 and BCL-XL [73,74]. Indeed, the spatiotemporal activity of MCL-1 is closely regulated through post-translational modifications in a highly cell- and context-specific manner. A series of enzymes have been implicated in the post-translational control of MCL-1, including extracellular signal-regulated kinase (ERK), glycogen synthase kinase 3 (GSK-3), and CDK1, which have been reported to phosphorylate at least one of 10 residues in the MCL-1 PEST region. ERK-mediated phosphorylation enhances the stability of MCL-1 and appears to confer chemo-resistance in breast cancer cell lines, whereas CDK1-mediated phosphorylation targets MCL-1 for degradation during mitotic arrest, and GSK-3-mediated phosphorylation targets MCL-1 for proteosome-mediated degradation [75–78]. Furthermore, GSK-3 protein expression is inversely correlated with *MCL1* expression in primary breast cancer patient tissue, and increased levels of MCL-1 protein in this patient cohort are associated with worse prognosis [79].

Multiple E3 ubiquitin ligases, including SCFβ-TrCP, FBW7, TRIM17, APC/CCdc20 and MULE (MCL-1 ubiquitin ligase E3), have been shown to link ubiquitin chains to MCL-1 in a phosphorylation – dependent or – independent manner to target MCL-1 for degradation [52]. The relationship between MULE and MCL-1 is particularly interesting as MULE contains a BH3 domain that specifically interacts with MCL-1, and this interaction is abrogated when MCL-1 is occupied by BIM [80,81]. MCL-1 protein becomes stabilized upon MULE knock-down and this increases resistance to DNA-damaging agents [80]. Both loss-of-function mutations and promoter hypermethylation of the E3 ubiquitin ligase FBW7 have been identified in breast tumors suggesting that the extent of MCL-1 protein upregulation in breast cancer may be as yet underappreciated [82,83].

A series of deubiquitinases (DUBs) functionally oppose E3 ligases by removing ubiquitin chains and have been reported to stabilize MCL-1 in a range of cancer types. These include Ku70, USP13, DUB3 (through MGMT), JOSD1, and USP9X, although specific roles in regulating MCL-1 during breast tumourigenesis have yet to be defined [84–89]. Importantly, suppression of DUBs upstream of MCL-1 is an attractive approach by which MCL-1 levels could be decreased in cancers (Figure 3), although the principal enzymes responsible for MCL-1 stabilization are highly cell-type specific and dependent on the wider proteomic profile of the target cell [90].

Indirect inhibition of MCL-1 may also be achievable through targeted degradation by a novel class of therapeutic agents termed proteolysis-targeting chimeras (PROTACs). These hetero-bifunctional compounds harness endogenous degradation machinery by tagging a target protein with a linker group to promote interaction with a specific E3 ubiquitin ligase. In the case of MCL-1, it was recently demonstrated that the dMCL1-2 PROTACs, composed of the Cullin4A (CUL4A) -DNA-binding protein 1 (DDB1)- Cereblon (CRBN) E3 ubiquitin ligase complex, could effectively degrade MCL-1 and activate apoptosis, as evidenced by a concomitant increase of cleaved-caspase-3 [91,92].

Therefore, MCL-1 is tightly regulated at every step from gene expression to protein degradation, and the mechanisms involved may be harnessed to indirectly inhibit MCL-1 function during breast tumorigenesis, or serve as biomarkers for patients who could benefit from MCL-1 inhibition (Figure 3).

## Pharmaceutical targeting of MCL-1 with BH3-mimetics

During tissue homeostasis, various stress or developmental stimuli trigger alterations in the balance of proteins within the BCL-2 family. This can enable pro-apoptotic proteins to overwhelm pro-survival proteins and exceed the required threshold to execute apoptosis (Figure 1). For example, P53 activation in response to DNA damaging agents such as γ-irradiation, etoposide or adriamycin, transcriptionally upregulates expression of pro-apoptotic BH3-only proteins to mediate DNA-damage induced apoptosis [93–95]. However, these mechanisms are often deregulated in cancer where induction of pro-apoptotic BH3-only protein expression may fail and/or pro-survival BCL-2 family proteins are elevated, and the threshold for triggering apoptosis is not attained despite the receipt of significant cellular stress. As many of the upstream pathways that regulate expression of the BCL-2 family are disrupted in cancer, direct targeting of pro-survival BCL-2 proteins is an attractive way to restore apoptosis.

A class of drugs termed BH3-mimetics have been developed to phenocopy the function of BH3-only proteins by occupying the BH3-binding groove of pro-survival BCL-2 family proteins, liberating pro-apoptotic proteins to enable activation of BAX/BAK. The first BH3-mimetics, ABT737 and the orally bioavailable derivative ABT263 (navitoclax), mimic the BH3-only protein BAD by binding to BCL-2, BCL-XL, and BCL-W, and effectively induce apoptosis in cells which are dependent upon these pro-survival family members [96,97]. Clinical use of navitoclax has been limited due to thrombocytopenia, as platelets are heavily dependent on BCL-XL, but careful dosing can limit this side-effect and numerous clinical trials are investigating navitoclax as a potential combination agent for the treatment of solid cancers [98,99] (clinicaltrials.gov). Inhibition of BCL-2/BCL-XL has shown anti-cancer function in preclinical models of breast cancer where navitoclax inhibits TNBC patient derived xenograft (PDX) growth when administered in combination with docetaxel [100]. Furthermore, an additional BCL-2/BCL-XL-specific BH3-mimetic that may avoid thrombocytopenia, called AZD0466, has recently entered clinical investigation [101].

The first clinically approved BH3-mimetic, ABT199 (venetoclax), specifically targets BCL-2 and is currently used in chronic lymphocytic leukemia (CLL) and AML, which are often dependent upon BCL-2 for cancer cell survival [102–104]. *BCL2* is an ER target gene commonly upregulated in ER-positive breast cancer, and venetoclax enhances the response of ER-positive PDX models to hormone therapy [105,106]. Initial clinical evaluation of ventoclax in solid tumors was conducted in combination with tamoxifen to treat metastatic ER-positive breast cancers expressing BCL-2, where it was well tolerated and showed promising anti-cancer effect [107]. The addition of venetoclax also enhanced the anti-tumor effect of fulvestrant and palbociclib (CDK4/6 inhibitor) in preclinical ER-positive breast cancer models, but a more complete assessment of the clinical value remains ongoing [108,109].

In recent years huge progress has been achieved in the development of MCL-1-specific BH3-mimetics with five independent compounds currently undergoing clinical evaluation in hematologic malignancies, and recruitment is underway for a further clinical trial in solid tumors, including breast cancer (NCT04837677) (Table 1). Whilst detail of the specificity and direct pro-apoptotic impact of some of these drugs is currently unknown, preclinical evidence strongly indicates that MCL-1 can be effectively inhibited to confer an anti-cancer effect *in vitro* and *in vivo*. Indeed, S63845 (a tool compound related to MIK665/S64315), AMG-176, AZD5991, and VU661013 effectively induce apoptosis in AML and MM cells *in vitro* and in xenograft mouse models, with clear synergy in combination with BCL-2 inhibition [110–115]. Importantly, combinatorial targeting of BCL-2 and MCL-1 is tolerated in mice and is under investigation in clinical trials of hematological cancers (NCT03672695, NCT04702425) [111–114].

**Table 1.** Clinical trials with MCL-1 specific BH3 mimetics. Data extracted from ClinicalTrials.gov.

Compound	Combination	Disease	Phase	Status	ID/Reference
MIK665/ S64315	-	Lymphona/ MM	I	Completed	NCT02992483
MIK665/ S64315	-	MM/MDS	I	Completed	NCT02979366
MIK665/ S64315	VOB560	Non-Hodgkin lymphoma/ AML/MM	I	Recruiting	NCT04702425
MIK665/ S64315	Azacitidine	AML	I/II	Recruiting	NCT04629443
MIK665/ S64315	Venetoclax	AML	I	Recruiting	NCT03672695
AMG176	Azacitidine/ Itraconazole	MM/AML	I	Recruiting	NCT02675452
AMG176	Venetoclax	Hematologic malignancies	I	Terminated (safety)	NCT03797261
AMG397	Dexamethasone/ Azacitidine	Hematologic malignancies	I	Terminated (strategic, not safety)	NCT03465540
AZD5991	Venetoclax	Hematologic malignancies	I/II	Suspended	NCT03218683
AZD5991	Azacitidine	AML	I	Recruiting	NCT03013998
ABBV467	-	MM	I	Terminated (strategic considerations)	NCT04178902
PRT1419	-	Hematologic malignancies	I	Recruiting	NCT04543305
PRT1419	-	Advanced solid tumors, including breast	I	Recruiting	NCT04837677

As well as successfully inducing apoptosis in several models of hematological malignancies, subsets of breast cancer cells are sensitive to MCL-1-specific BH3-mimetics *in vitro* [110–112,115,116]. For example, S63845 improves the response of the BT-474 *HER2*-amplified breast cancer cell line to lapatinib (HER2 tyrosine kinase inhibitor) in 2-D cell culture, and also inhibits growth of TNBC PDX cells in 3-D tumoursphere assays [110,117]. Further, it has been demonstrated that S63845 can successfully induce apoptosis when used in combination with either lapatinib, docetaxel, or trastuzumab (HER2-targeting monoclonal antibody) in another *HER2*-amplified breast cancer cell line, SK-BR-3, through liberation of pro-apoptotic BH3-only activator BIM [117].

*In vivo*, S63845 illustrates single agent efficacy in restricting tumor growth in the *MMTV-PyMT* mouse model of breast cancer [118]. Combining S63845 with docetaxel in two TNBC PDX mouse models, or trastuzumab in a *HER2*-amplified PDX mouse model, has been shown to significantly impair tumor growth and prolong survival, although monotherapy with the MCL-1 inhibitor failed to markedly prevent tumor growth [117]. Similar results have been documented in a *BRCA1*-mutant TNBC PDX mouse model, where S63845 synergized with the PARP inhibitor olaparib [124]. Finally, single-agent treatment with VU661013 effectively inhibited growth of TNBC xenografts (using HCC1187 and BT20 cell lines) and sensitized tumors to treatment with either docetaxel or doxorubicin [115]. Taken together, these findings suggest that the most potent effects of MCL-1 inhibition in breast cancer may occur when used in combination with additional therapies that drive an increasingly “primed” apoptotic state [16].

## MCL-1 is required for breast cancer cell survival

The prevalence of MCL-1 upregulation in breast cancer suggests that it is required for tumor development, and associations between high MCL-1 and poor outcome may also indicate a role for MCL-1 in resistance to existing treatment regimen. Similar to the broad range of MCL-1 expression observed in breast cancer epithelial cells, human breast cancer cell lines representative of the major subtypes of disease express variable levels of MCL-1 and have provided a useful tool for assessing the essentiality of MCL-1 function in breast cancer [45,116,117,119,120]. Indeed, studies utilizing breast cancer cell lines first indicated a requirement for MCL-1 to maintain cell viability, as genetic knock-down of *MCL1* induces cell death in a subset of both TNBC and ER-positive breast cancer cell lines grown in 2-D monolayer [119,121,122]. Whilst knock-down of *MCL1* alone is sufficient to induce apoptosis in some TNBC and ER-positive breast cancer cell lines, functional buffering by BCL-XL can confer resistance to MCL-1 loss [119,121,122]. This is in line with the analysis of breast cancer cell lines in Cancer Cell Line Encyclopedia data, which highlights a role for both *MCL1* and *BCL2L1* (BCL-XL) in maintenance of breast cancer cell line viability in 2-D culture, whereas *BCL2* appears largely non-essential in this context (Figure 2) [123,125,126].

The requirement for MCL-1 is exacerbated during 3-D tumoursphere growth in non-adherent culture conditions, where the functional redundancy often witnessed between MCL-1 and BCL-XL in 2-D monolayer growth is less profound [118]. Genetic knock-down or pharmacological blockade of MCL-1 also restricts the growth of TNBC cells (MDA-MB-468) in xenograft mouse models [45,120]. It has been illustrated that the decreased viability upon MCL-1 knock-down/knock-out or pharmacological inhibition in breast cancer cell lines is associated with redistribution of BIM and the subsequent induction of apoptosis, as evidenced by cytochrome *c* release, cleavage of caspase-3 and/or PARP, whilst pan-caspase inhibition (with Q-VD-OPh) or deletion of the intrinsic apoptotic effectors BAX/BAK alleviates this effect [45,115,116,118,119,122,127].

Alongside the requirement of MCL-1 for breast tumor growth, MCL-1 has also been implicated in acquired resistance to tamoxifen or fulvestrant treatment in ER-positive breast cancer cell lines [128]. Furthermore, MCL-1 protein is elevated in post-chemotherapy biopsies of TNBC patients and increased MCL-1 has been shown in multiple *in vitro* models of acquired paclitaxel resistance, where knock-down of *MCL1* restores the apoptotic response [47–49]. MCL-1 has also emerged as an important resistance factor to BCL-2/BCL-XL specific BH3-mimetics in hematopoietic cancers, encouraging the investigation of combinatorial treatments to also target MCL-1. This could have relevance in ER-positive breast cancer where venetoclax is currently undergoing clinical trials, as MCL-1 expression is associated with resistance to BCL-2/BCL-XL inhibition in ER-positive breast cancer cell lines. Indeed, targeting of MCL-1 has been shown to sensitize cells and effectively inhibit tumor growth *in vitro* and *in vivo* when administered in combination with BCL-2/BCL-XL inhibitors [122,127]. Importantly, ER-positive breast cancer cell lines adapt to MCL-1 or BCL-XL inhibition through BIM sequestration by the respective uninhibited pro-survival BCL-2 family protein, and this could be harnessed therapeutically through sequential treatment to liberate BIM and stimulate activation of BAX/BAK [129]. Interestingly, mono-therapeutic treatment with the MCL-1-specific BH3-mimetic VU661013 slowed the growth of HCC1187 and BT20 TNBC xenografts, and co-treatment with docetaxel or doxorubicin further enhanced the inhibition of tumor growth [115].

Taken together, this indicates that MCL-1 inhibition can sensitize different breast cancer subtypes to a range of therapies, though it is likely that refined treatment strategies will be required to circumvent significant toxicity in normal tissues. For example, MCL-1 is known to be important during homeostatic cardiomyocyte functionality [7,8,130]. Whilst the pro-tumor role of MCL-1 in models of breast cancer is highly reliant on BH3-dependent functions of MCL-1, there may be important non-apoptotic roles for MCL-1 in tissues outside the breast and the ability of MCL-1-targeting strategies to impact or spare these alternative functions must be considered given the fatality of complete *Mcl1* deletion in mice [6,118,131]. Potential toxicity could be countered using antibody-drug conjugates (ADCs) to selectively target tumor cells. Indeed, targeted inhibition of BCL-XL with Mirzotamab clezutoclax (ABBV-155), composed of a BCL-XL inhibitor linked to a monoclonal anti-B7H3 antibody, is currently undergoing phase I clinical trials for the treatment of B7H3-expressing relapsed/refractory solid tumors (NCT03595059) [132].

As well as the documented tumor cell-intrinsic role for MCL-1, tumor explant and co-culture experiments have also highlighted a pro-tumor function for MCL-1 within the stromal microenvironment. Breast cancer associated fibroblasts (bCAFs) are dependent upon MCL-1 for survival and through secretion of IL-6, bCAFs can induce upregulation of *MCL1* mRNA and protein in luminal breast cancer cells to reduce sensitivity to BCL-2/XL inhibition [133]. Genetically engineered mouse models (GEMM) allow investigation of tumor cells within an intact tumor microenvironment and have revealed a key role for MCL-1 in tumor development and maintenance of mammary cancer *in vivo* (Table 2). The *MMTV-PyMT* mouse is molecularly and histopathologically identified as representing human luminal B breast tumors and faithfully recapitulates the tumorigenic process from hyperplasia to invasion of the lung and lymph nodes [134,135]. Genetic deletion of *Mcl1* in the mammary epithelium of *MMTV-PyMT* mice reveals an absolute requirement for MCL-1 in mammary tumor development and outgrowth [45]. Dramatically, when *Mcl1* is conditionally ablated in the tumor epithelium of mice possessing allografted mammary tumors 8/9 tumors regressed, and in 4/9 cases this regression was complete and underpinned long-term tumor-free survival [118]. These genetic studies reveal an exquisite dependency of *MMTV-PyMT* mammary tumors on MCL-1 for tumor maintenance and development (Table 2).

**Table 2.** Summary of genetically engineered mouse models revealing a requirement for *Mcl1* in mammary cancer.

Oncogenic driver	*Mcl1* alteration	Effect
*MMTV-PyMT*	Deletion in mammary epithelium (*MMTV-Cre;Mcl1^fl/fl^)*	Outgrowth of tumors that have escaped *Mcl1* deletion 45
*MMTV-PyMT*	Whole body heterozygous deletion once tumors established (*Rosa-Cre^ER^;Mcl1^fl/+^)*	Delayed tumor growth 118
*MMTV-PyMT*	Conditional deletion in tumor fragment transplant once tumors established (*Rosa-Cre^ER^;Mcl1^fl/fl^)*	Tumor regression, long term tumor free survival in almost 50% of mice 118
*Brca1^fl/fl^;Trp53^fl/fl^*	Over-expression via intraductal injection (Lenti-*Mcl1_P2A_Cre*)	Accelerated tumor development (survival 180d v 238d) 124
*Brca1^fl/fl^;Trp53^fl/fl^;Col1a1^invCAG-^^Myc^^−^^IRES^^−^^Luc^^/+^*	Over-expression via intraductal injection (Lenti-*Mcl1_P2A_Cre*)	Accelerated tumor development (survival 70d v 126d) 119

The importance of MCL-1 in breast cancer has also emerged from new models that recapitulate the genetic events in *BRCA1*-mutant tumors, where loss-of-function mutations in *TP53* and amplification of *MYC* occur in 65% and 44% of cases, respectively [124]. In elegant mouse models, deletion of *Brca1* and *Trp53*, alongside concurrent amplification of *MYC*, results in mammary tumors that molecularly resemble basal-subtype human breast cancers. Comparative oncogenomics of these mouse tumors and human breast cancers identifies common amplification of the *MCL1* locus, and MCL-1 overexpression was shown to accelerate mammary tumor development in this mouse model (Table 2). Knock-down of *Mcl1* in GEMM-derived organoids further highlighted a requirement for MCL-1 expression, thus confirming the necessity of MCL-1 in a genetic model of *BRCA1*-mutant basal-like TNBC [124]. In line with these studies, which demonstrate that genetic manipulation of *MCL1* alters tumor development, pharmaceutical targeting of MCL-1 with the BH3-mimetic S63845 can also inhibit growth of established *MMTV-PyMT* tumors in a BAX/BAK-dependent manner, indicating on-target efficacy of pharmacological inhibition of the canonical MCL-1 function to inhibit mammary tumor growth [118].

PDX models of TNBC have shown that a reversible cell state enriched in a cancer stem-like cell (CSC) gene expression signature arises in drug-refractory breast cancer, and as elevation of MCL-1 occurs in treatment-resistant samples it is tempting to speculate that MCL-1 may facilitate this process [48,49,136,137]. Indeed, knock-down of *MCL1* can restrict tumor initiation *in vivo* when tested in limiting dilution transplantation assays with the SUM159PT TNBC cell line, whilst MCL-1 overexpression increases the stemness of MDA-MB-468 cells (TNBC) as determined by an increased CD44^hi^/CD24^lo^ cell-surface profile and enhanced tumorsphere-forming capacity [49]. Furthermore, analysis of extensive breast cancer datasets reveals an association between *MCL1* expression and markers of both stemness and an epithelial-to-mesenchymal transition (EMT) [118].

It has been suggested that the primary function of MCL-1 in breast CSCs is due to non-canonical activity at the inner mitochondrial membrane where it functionally cooperates with MYC to elevate mitochondrial biogenesis and oxidative phosphorylation, although this non-canonical function was not disrupted when MCL-1 was inhibited pharmacologically using the VU0659158 BH3-mimetic in MDA-MB-436 or SUM159PT TNBC cell lines [49]. Recent work in our group suggests that a key function of MCL-1 in conferring stem-like behavior in breast cancer is through its canonical role within the BCL-2 family. We demonstrated that deletion or pharmacological inhibition of MCL-1 with either S63845 or A1210477 BH3-mimetics impaired the tumorsphere-forming capacity of breast cancer cells, and that genetic ablation of pro-apoptotic effector proteins BAX and BAK abrogates this effect [118]. There are subtle differences in the interactions between MCL-1 and distinct BH3-mimetics, and a more complete characterization of the functional consequence of each MCL-1 inhibitor is required [115]. For effective anti-cancer therapy, BH3-mimetic drugs must counteract the pro-tumor functions of MCL-1 that occur *in vivo*. Promisingly, S63845 effectively mimics the anti-tumor impact of *MCL1* deletion in a mouse model of breast cancer and the restricted tumor growth was dependent upon the presence of BAX/BAK, suggesting that the major anti-apoptotic function of MCL-1 in breast cancer cell survival can be effectively targeted with BH3-mimetics [118]. Advances in BH3-profiling techniques have the potential to direct BH3 mimetic use in the clinic. Indeed, the ability of cancer therapeutics to prime individual cancers for apoptosis can be gauged *ex vivo* using an adapted BH3-profiling protocol [138]. The utility of inhibiting different pro-survival BCL-2 proteins in combination with chemotherapy has been demonstrated using freshly isolated non-small cell lung cancer patient samples [139]. It can be envisioned that such technologies could pave the way for personalized use of BH3 mimetics in breast cancer.

## Concluding remarks

Whilst preclinical work has validated MCL-1 as a relevant target in breast cancer, the essentiality of MCL-1 in many normal cell types may necessitate careful administration of MCL-1 targeting drugs to facilitate a therapeutic window. To achieve tumor-specific cell death, MCL-1 inhibition may work best as a combination therapy, where it could be employed to exacerbate the effect of conventional cytotoxic and targeted treatment approaches. Distinct BCL-2 family proteins may play differentially prominent roles in individual cancers, and biomarkers to reveal potential sensitivity to MCL-1 inhibition would allow effective tailoring of treatment. It is possible that inhibition of multiple pro-survival BCL-2 family proteins is required to efficiently restore apoptosis in some patients, and this provides a compelling argument for further research into understanding which subsets of patients could benefit from MCL-1-specific inhibition; the optimal timing for prospective treatment; the potential value of co-administration of additional therapies; and in-depth characterization of the bona fide role that MCL-1 plays in breast development and tumorigenesis.

## Data Availability

References are provided for publically available data that was accessed through cBioportal (https://www.cbioportal.org) and DepMap (https://depmap.org).
